# WatchPose: A View-Aware Approach for Camera Pose Data Collection in Industrial Environments

**DOI:** 10.3390/s20113045

**Published:** 2020-05-27

**Authors:** Cong Yang, Gilles Simon, John See, Marie-Odile Berger, Wenyong Wang

**Affiliations:** 1 MAGRIT Team, INRIA/LORIA, 54600 Nancy, France or cong.yang@uni-siegen.de (C.Y.); gilles.simon@loria.fr (G.S.); marie-odile.berger@loria.fr (M.-O.B.); 2 Faculty of Computing and Informatics, Multimedia University, Cyberjaya 63100, Selangor, Malaysia; johnsee@mmu.edu.my; 3 School of Information Science and Technology, Northeast Normal University, Changchun 130000, China

**Keywords:** data acquisition, augmented reality, pose estimation, deep learning, industrial environments

## Abstract

Collecting correlated scene images and camera poses is an essential step towards learning absolute camera pose regression models. While the acquisition of such data in living environments is relatively easy by following regular roads and paths, it is still a challenging task in constricted industrial environments. This is because industrial objects have varied sizes and inspections are usually carried out with non-constant motions. As a result, regression models are more sensitive to scene images with respect to viewpoints and distances. Motivated by this, we present a simple but efficient camera pose data collection method, WatchPose, to improve the generalization and robustness of camera pose regression models. Specifically, WatchPose tracks nested markers and visualizes viewpoints in an Augmented Reality- (AR) based manner to properly guide users to collect training data from broader camera-object distances and more diverse views around the objects. Experiments show that WatchPose can effectively improve the accuracy of existing camera pose regression models compared to the traditional data acquisition method. We also introduce a new dataset, Industrial10, to encourage the community to adapt camera pose regression methods for more complex environments.

## 1. Introduction

Camera pose (location and orientation) estimation is a fundamental task in Simultaneous Localization and Mapping (SLAM) and Augmented Reality (AR) applications [1,2,3]. Recently, end-to-end approaches based on convolutional neural networks have become popular [4,5,6,7,8,9,10,11,12,13]. Instead of using machine learning for only specific parts of the estimation pipeline [14,15,16,17], these methods aim to learn the full pipeline with a set of training images and their corresponding poses. Building on that, the trained models directly regress the camera pose from an input image. Several works [4,7] in the literature report that those methods are plausible in regular living environments (e.g., along the street or path), achieving around a 9∼25 m and 4∼17${}^{\circ }$ accuracy in localization and orientation, respectively.

**Figure 1:**
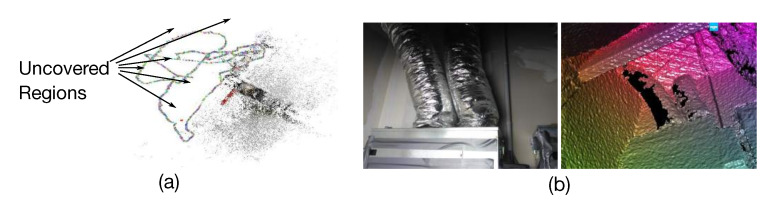
Challenges of collecting training data in industrial environments. (**a**) There are some uncovered regions within the training data using traditional video collection [6]. (**b**) There are some missing parts within the reconstructed 3D model on specular surfaces using Kinect Fusion [23,24].

**Figure 2:**
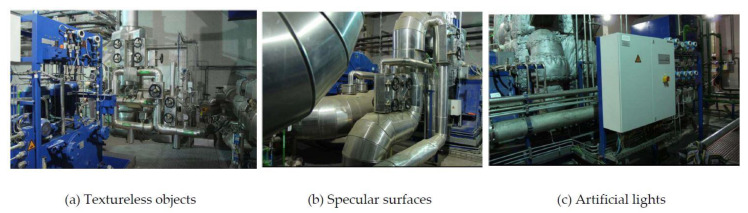
Sample images in industrial environments.

However, the utility of such methodology is limited in industrial environments. This is because people in such environments usually exhibit two kinds of typical motions: (1) They inspect industrial objects with non-constant motions and views and (2) they inspect industrial objects from different distances. For the first case, traditional data collection methods cannot cover enough viewpoints to properly train a generalized pose regression model. For example, in [5] a smartphone was used by a pedestrian to take videos around each scene while in [7,18], robot cars were designed to take pictures along the street. Meanwhile, viewpoints in industrial environments may be restricted to only random collection and limited moving directions. Figure 1a visualizes the viewpoints of the video collection method and it is obvious that most regions around the industrial object are uncovered. As a result, the trained models easily over-fit to such limited training data and may not generalize well to uncovered scenes. For the second case, a simple idea is to apply data augmentation techniques such as image zooming to imitate different camera-object distances (hereinafter is referred to as “camera distance”). However, over-zooming could reduce the quality of training data and decay the regression accuracy [19]. To solve these problems, one possible way is to reconstruct a 3D model of the target object and then generate training data via rendering [20,21,22]. Nevertheless, the problem we tackle here involves more complex scenarios: 3D object models are not always available in industrial environments and in many cases, they are hard to construct because of the presence of textureless and specular surfaces under sharp artificial lights [1]. Figure 1b shows an example of an industrial object and its reconstructed 3D model using Kinect Fusion [23,24]. We can easily observe the missing parts (the black holes) in the model. In fact, as shown in Figure 2, industrial environments are typically inundated with such textureless and occluded objects and specular surfaces, etc.

**Figure 3:**
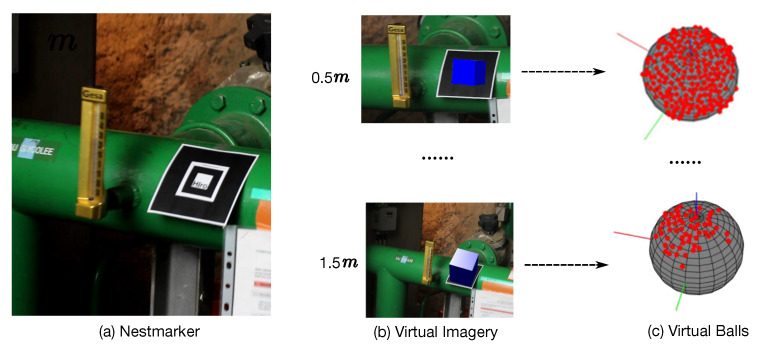
Main idea of WatchPose. (**a**) The Nestmarker is placed close to the target object. (**b**) A virtual imagery is drawn for checking the correctness of marker tracking (the blue box) from different camera distances. (**c**) For each camera distance interval, a virtual ball is drawn and automatically switched for visualizing the captured viewpoints (the red points) and navigating the uncovered regions.

Thus, we seek to find an efficient approach that can collect pose training data from sufficient viewpoints and camera distances. Towards this goal, as shown in Figure 3, a view-aware approach, WatchPose, is introduced. The basic idea of WatchPose is to place a marker close to the target object and the training data is collected through marker tracking [25,26] with dynamic viewpoint control around the object. Here, we introduce three strategies to improve the efficiency of this process: (1) We propose Nestmarker, a combination, and nesting of traditional markers, for marker tracking. The Nestmarker performs detection and tracking from different camera distances since it contains two markers with fixed relative positions and different sizes. Thus, we can flexibly collect training data from a range of larger and smaller camera distances as compared to traditional markers. (2) During the data collection process, virtual imagery is drawn on the marker for checking the correctness of marker tracking (the blue box). (3) A virtual ball is drawn for visualizing the captured viewpoints (the red points) and navigating the uncovered regions. With these strategies, our data collection approach is applied in a real-time setting and it is robust towards variations in objects, surfaces, and lights since the camera poses are directly generated from marker tracking. Moreover, it is easier for us to visually control the camera distances, viewpoint locations, and densities since this approach can navigate the camera controller (e.g., people, robot, etc.) to move the camera dynamically so that most regions around the object can be covered. Once the training data are collected, we apply a set of post-processing steps such as calibrating the camera poses from the marker to the target object, data augmentation, etc.

To encourage the community to adapt camera pose regression methods towards more complex domains, we collected a new benchmark dataset, Industrial10, to reflect the challenges of industrial environments. Industrial10 comprises of training and testing data of 10 industrial objects. To assess the efficiency of the proposed method, five actively used pose regression methods are employed in our experiments. Evaluations show that the proposed method can effectively improve the camera pose regression accuracy compared to the traditional data collection method.

The contributions of this work are summarized as follows: (1) We propose a novel training data collection method called WatchPose to improve the performance of camera pose regression in industrial environments. The proposed method is sufficiently general and can be extended to other scenarios. (2) We introduce a new dataset, Industrial10, to spur the computer vision systems community towards innovating and adapting existing camera pose regression approaches to cope with more complex environments.

## 2. Related Works

Here, we briefly glanced through several existing camera pose data collection strategies followed by a review of camera pose regression methods. For a more detailed treatment of this topic in general, the recent compilation by Shavit and Ferens [27] offers a sufficiently good review.

### 2.1. Camera Pose Data Collection

In general, camera pose collection methods can be classified into two ways: Direct and indirect approaches. With direct approaches, camera poses are acquired from markers or physical sensors. For example, Brachmann et al. [28] collected the ground-truth camera poses via integrating a set of traditional markers densely surrounding the target object. Tiebe et al. [29] collected the camera poses via a finely-controlled robot arm. In most cases, camera poses are collected indirectly. For instance, the actively used dataset 7Scenes [30] was first recorded from a handheld Kinect RGB-D camera [31]. After that, the ground-truth camera poses were extracted by Kinect Fusion technique [24]. To generate the Cambridge Landmarks dataset, Kendall et al. [5] first captured high definition videos from around each scene using a smartphone and then proceeded to extract ground-truth camera poses using Structure from Motion (SfM) techniques [32]. Alternatively, Wohlhart et al. [20] first reconstructed 3D models of the target objects and then extracted training data (images and their pose annotations) by rendering the 3D models. More recently, the WoodScape [18] dataset were collected from a car mounted with a set of sensors (e.g., Inertial Measurement Unit,GPS, LiDAR, multiple cameras, etc.) along selected streets in USA, Europe, and China. Camera poses of this dataset could be extracted by fusion of LiDAR, IMU, and camera sensors. However, these indirect collection methods cannot be properly applied in industrial environments since industrial objects are normally textureless and may possess specular surfaces under strong artificial lights. For direct methods, it is also not feasible to use traditional markers or robot arms as well. This is because in addition to high costs, there are normally limited spaces around industrial objects and people often inspect them from varying distances. Therefore, we introduce WatchPose, which can better imitate watchers’ motion and avoid the aforementioned problems. WatchPose extends the idea of traditional markers [28] to support data collection from both close and far distances. Moreover, it is robust to textureless and specular surfaces since the camera poses are directly collected from Nestmarker. Finally, as more viewpoints are covered in the data collection of WatchPose, the trained models can generalize more robustly.

### 2.2. Camera Pose Estimation

Leveraging on the idea of transfer learning, more and more researchers attempt to learn models for pose estimation tasks. Generally, these methods function by training descriptors, classifiers, and regressors using a variety of ways. For descriptors, Gu et al. [33] built discriminative models by training a mixture of HOG templates, while Aubry et al. [34] employed them for 3D object detection and pose estimation. In contrast to mixed descriptors, Masci et al. [35] trained a single-layer neural network with different hashing approaches to compute discriminative descriptors for omnidirectional image matching. Wohlhart et al. [20] extend this idea further and train a LeNet [36] to compute features for rendered object views that capture both the object identity and camera poses. Though these approaches can efficiently handle a large number of objects under a large range of poses, they highly rely on handcrafted representations [33,34] and 3D object models [20,21,22]. For classifiers, Schwarz et al. [22] computed image features by a pre-trained Convolutional Neural Network (CNN) and then fed them to a Support Vector Machine (SVM) to determine object class, instance, and pose. Brachmann et al. [28,37] employed image features from [30] to train a random forest for object detection, tracking, and pose estimation. Those approaches achieved promising performance in cluttered scenes, but they normally require additional reconstruction steps to generate dense scenes and object models. For regressions, Shotton et al. [30] introduced a regression forest that is capable of inferring an estimate of each pixel’s correspondence from a given image to 3D points in the scene’s world coordinate frame. With this, the computation of feature descriptors are not required. Unlike their approach, Gupta et al. [38] proposed a 3-layer convolutional neural network to regress coarse poses using detected and segmented object instances. However, these algorithms are constrained by the use of RGB-D images to generate the scene coordinate label, which in practice limits its use in industrial environments. To improve it, Kendall et al. [5,6] proposed PoseNet that directly regresses the camera pose from RGB images. However, it easily overfitted with its training data while its localization error on indoor and outdoor datasets was an order of magnitude larger. Such limitations motivated a surge of absolute pose estimation methods such as Bayesian PoseNet [39], MapNet [7], LSTM-Pose [40], Hourglass-Pose [8], SVS-Pose [9], BranchNet [10], and NNnet [11] to involve deeper encoders, better loss functions, and more sensor data. However, as discussed in [4], those pose regression models is more closely related to pose approximation via image retrieval. In other words, the training data should cover viewpoints and camera-distances as much as possible. Thus, we try to improve the performance of pose regression models by feeding better data. Experiments in Section 4 showed that performance improvement with better training data is more obvious, and particularly suitable for industrial type of objects.

## 3. WatchPose

WatchPose employs a Nestmarker-based strategy to generate training images and their camera pose labels built on AR frameworks [1]. The reason is that AR frameworks have powerful libraries for robust and real-time marker tracking [25]. In other words, we can directly collect accurate camera poses during the marker tracking process. The proposed pose generation strategy is general and can be built on top of most existing pose training and estimation measures.

### 3.1. Nestmarker

**Figure 4:**
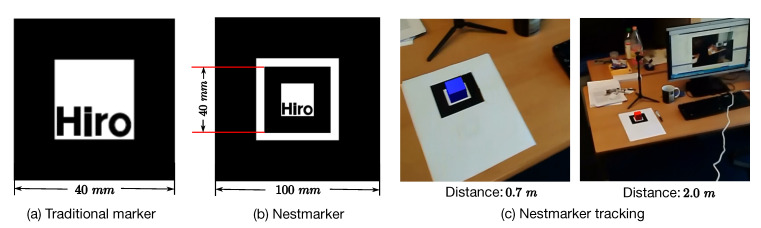
The Nestmarker and its tracking system.

Inspired by [41], Nestmarker is a combination and nesting of traditional markers (Figure 4). In particular, the inner 50% of the Nestmarker is a traditional 40 mm square marker plus a 10 mm white square gap. We call this inside marker as the small marker. Using it as a pattern, we add a continuous border to make a 100 mm square marker (big marker). In other words, a Nestmarker contains two square markers: A small marker and a big marker. Hence, the proposed Nestmarker has three distinct features: (1) Since patterns of small and big markers are rotationally asymmetric, both markers can be trained and tracked independently. (2) By marker tracking, the collected camera poses from small and big markers are correlated since their relative positions are fixed and their sizes are known. (3) The big marker can be tracked from larger camera distances than the small marker. Based on our preliminary experiment using a 1080P HD Webcam and 1024×768 frame resolution, the small and big markers could be detected between 5 cm to 80 cm and 13 cm to 300 cm camera distances, respectively. With these features, we developed a Nestmarker tracking system as illustrated in Figure 4c. Particularly, the big marker is triggered and tracked (e.g., red box) when the small marker cannot be detected due to big camera distances. Otherwise, the small marker is triggered and tracked (e.g., blue box). In both cases, the square marker, from the black continuous border to the inner part, is tracked.

### 3.2. Data Collection

**Figure 5:**
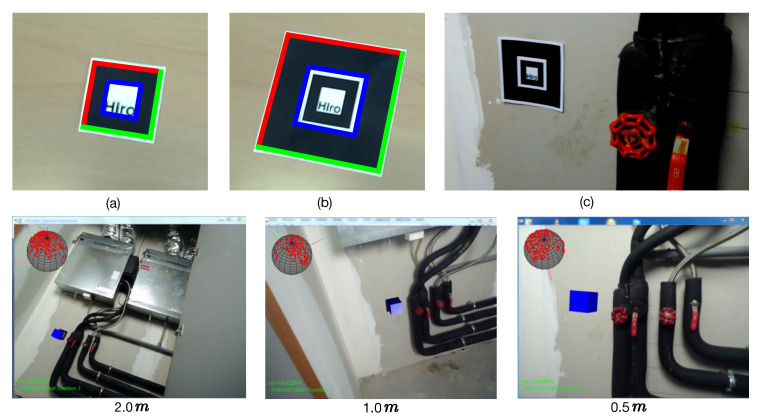
Camera pose training data collection using WatchPose.

In our work, the ARToolKit [25] library is employed for Nestmarker learning, tracking, and camera pose collection. Figure 5 shows an overview of our strategy. Firstly, the small and big marker patterns (Figure 5a,b) are independently learned by our system thereby they can be detected in the next steps. After that, the Nestmarker is placed close to an object (Figure 5c) and is tracked by a camera around the object. During tracking (the second row in Figure 5), a virtual image (blue box) is synchronously drawn above the marker so that we can visually check whether the marker has been detected. If the marker is correctly detected from the camera, a viewpoint (shown with red point) is simultaneously plotted on the virtual ball and the current image and the real camera pose are saved. In some cases, the collected image could be badly blurred due to fast camera motion or out-of-focus condition. For this, we quantitatively evaluate its blur effect based on the method in [42] and discard the image and its camera pose if its blur value exceeds a predefined threshold ϱ, which is set to 0.5 in our experiments.

During the capturing process, the virtual ball is automatically switched and rolled depending on the camera position and orientation. This strategy can effectively navigate the camera controller by determining the direction that the camera should be moved in order to capture training data from uncovered regions and distances. The pose computed using our algorithm is expressed in the Marker Coordinate System (MCS). The labels and/or added graphics must be expressed in this frame. To assist in this placement of the virtual scene in the MCS, reference points representing the object may be added in that frame. To do this, two views of the object with the marker are all that is needed. Using the marker, the poses for both views can be calculated and the 3D reference points can be reconstructed in the MCS by triangulation [43]. Pairing the points between the two views can be done manually if a few reference points are sufficient, or with the help of a Scale-invariant Feature Transform (SIFT)-like descriptor [44] if a denser point cloud is desired.

**Figure 6:**
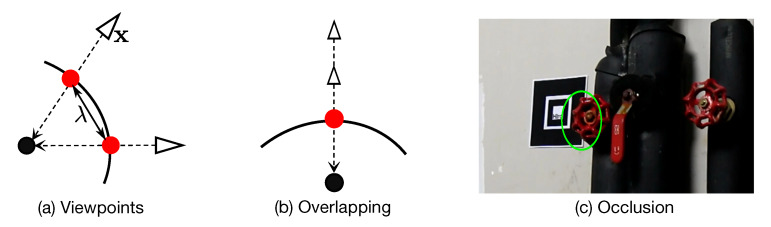
Viewpoint calculation and density control.

In order to properly plot viewpoints on the virtual ball, as shown in Figure 6a, a viewpoint (red point) is mapped to the virtual ball surface (arc) from a real camera position (triangle) with respect to the ball center point (black point). In our case, the real 3D camera position x is calculated by $x=-R^{\;T}*T$, where $R^{\;T}$ is a 3×3 camera rotation matrix and *T* is a 1×3 camera translation vector that are both directly generated in our system based on the ARToolKit library.

As shown in Figure 6a, we introduce a threshold λ to control the minimal viewpoint distance. With this, we can dynamically control the viewpoint distribution density by changing the λ value. In other words, if the current viewpoint is too close to a plotted one based on the threshold λ (i.e., <λ), the system will discard the current camera pose. It should be noted that there is a high probability that viewpoints from different camera positions could overlap on the virtual ball (Figure 6b) if they have the same orientation and their locations are on the same line with respect to the object. As such, we use multiple virtual balls to visualize and control the viewpoint distribution based on real camera distance intervals (Figure 5). In this work, we predefine 3 camera distances: 0.5 m, 1.0 m, and 2.0 m to control and visualize viewpoint distributions separately. For the overlapping and uncovered viewpoints within other distances (e.g., 0.3 m, 1.3 m, etc.), we introduce a data augmentation strategy, described in Section 3.3, to enrich the collected data. There is another possibility that the Nestmarker may be occluded by the object under certain viewpoints thereby it cannot be properly detected (Figure 6c). In this case, the small marker will be triggered if the big marker is occluded. If both markers are occluded, we can place multiple markers around the object in advance depending on scenarios. In our case, we only place another small marker close to the object to avoid occlusion since this phenomenon normally appears when the camera is close to an object.

**Figure 7:**
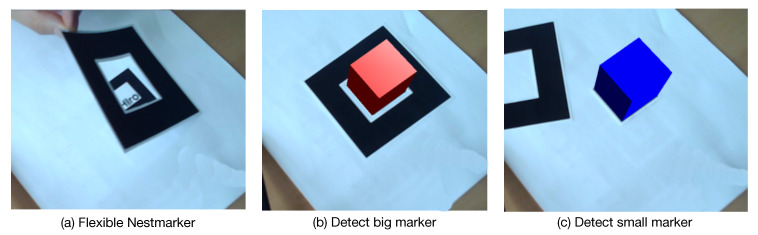
Flexible use of Nestmarker in narrow environments.

In practise, some industrial objects may have limited space (e.g., in the corner or occluded) for data collection. In such a case, as an example shown in Figure 7a, a flexible Nestmarker is proposed in which the black continuous border and the nested small marker are detachable. In particular, we first capture training data from large camera distances using the large marker (Figure 7b). After that, the black continuous border is removed and only the small marker is kept for capturing training data from smaller camera distances (such as 5 cm to 80 cm in our case) (Figure 7c). Using this strategy, the proportion of the marker in an image can remain small.

### 3.3. Parametrization and Augmentation

For a collected camera pose p, we parameterize it with a 7-dimensional pose vector p=[x,q], where x contains 3 values representing the camera position while quaternion q contains 4 values representing the camera orientation. Here, the quaternion q can be directly converted from the camera rotation matrix *R*. Consequently, for each collected image *I*, a 7-dimensional p is constructed to represent the camera pose of *I*. We also apply an inpainting process using Coherency Sensitive Hashing (CSH) [45] to remove the marker from each image. The main reason is that in practice there is a time distance between learning and testing ages. It is quite normal to have a marker during the learning age and to remove it during the application time. CSH relies on hashing to seed the initial local matching and then on image coherence to propagate good matches. As a marker location is automatically detected and saved by ARToolKit, CSH can quickly find matched parts in the marker neighbourhood in the image plane and thereby replace the marker region. Moreover, to reduce the inpainting error, markers can be placed in the place with a homogeneous color or texture. This is not challenging in industrial environments since most walls or surfaces are painted with monotonous colors.

**Figure 8:**
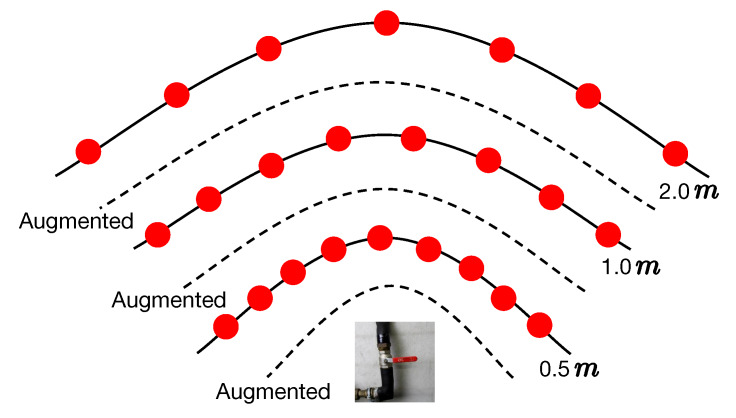
Camera pose augmentation.

After inpainting, we apply a data augmentation process to imitate different camera locations and orientations (Figure 8). We enrich each collected image by employing rotation and slight zooming (i.e., scaling along the camera axis). It should be noted that the number of times zooming is applied within a particular camera distance is highly dependent on the scenario. For instance, our experiment in Section 4.3.2 suggests that augmenting four times within a camera distance is enough to achieve promising pose estimation accuracy. Thus, if the training data are densely captured (with small distance intervals), the number of times augmentation is needed can be reduced or even cancelled altogether. To properly incorporate viewpoint density control and augmentation, we suggest that the viewpoint density λ (Figure 6a) should be reduced when the camera is closer to the object so as to avoid redundant images resulting from augmentation.

## 4. Experiments

In this section, we first introduce the proposed Industrial10 dataset and its properties. Built on that, we evaluate the WatchPose and widely used pose regression approaches.

### 4.1. Industrial10

**Figure 9:**
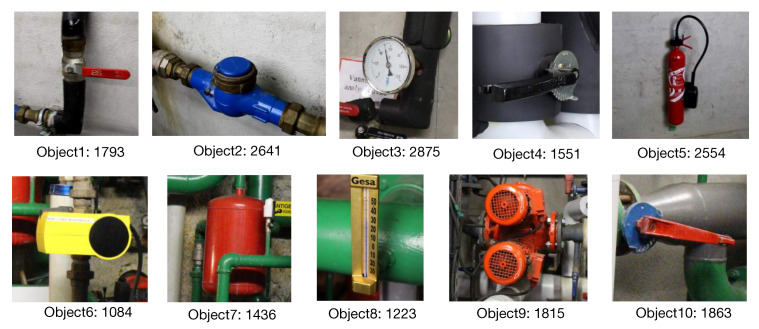
Sample images of 10 objects in industrial environments.

**Figure 10:**
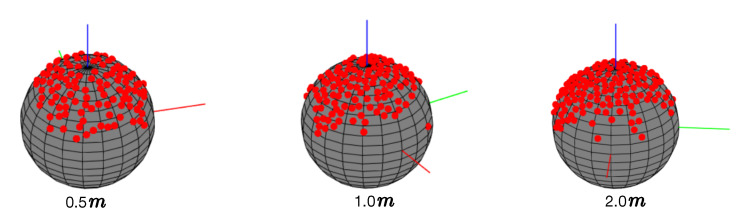
Viewpoints of Object1 in different camera distances.

To the best of our knowledge, there is no existing dataset specially designed for evaluating camera pose estimation in industrial environments. For this, we collected a dataset containing 10 industrial objects using our proposed WatchPose approach. As shown in Figure 9, the main purpose of selecting these objects is to reflect the real challenges from industrial environments. For example, Object1 and Object10 have similar appearances under certain viewpoints that may confuse object detection algorithms. Object6 has limited training data since it is located in a narrow environment. Since Object9 is relatively big, only part of its appearance was captured with small camera distances. Object7 is occluded by a green pipe so its appearance could look different depending on camera pose. For each object, we collected the training data from three camera distances: 0.5, 1.0, and 2.0 m (Figure 8) using the proposed WatchPose approach. Figure 10 presents an example depicting the viewpoint distributions of Object1 with different camera distances. We can observe that most regions around Object1 are covered. The number of original training data for the 10 objects is also shown in Figure 9 after their respective names. For testing, each object has a set of 200 testing images that are randomly collected within 0.5 to 2.0 m camera distances. The Industrial10 dataset is publicly available to the community (Please check congyang.de for more details).

In addition to the Industrial10 training data collected with WatchPose (named Industrial10-WatchPose), we also extracted another set of training data, named Industrial10-Traditional, using the traditional data acquisition approach. Specifically, Industrial10-Traditional was collected by capturing a high definition video around each object from camera distances 0.5, 1.0, and 2.0 m. In total, 3×10 videos were recorded and each video was then sub-sampled at 2Hz to generate its frames. These images are then inputted to the SfM pipeline to extract the camera poses. For fair comparison, two post-processing steps were followed: (1) We converted the camera poses from SfM to the same format as of Industrial10-WatchPose based on the pre-measured datum line. (2) We uniformly selected the same number of training images according to the training data distribution in Industrial10-WatchPose. For example, Object1 has 1793 training images in both Industrial10-WatchPose and Industrial10-Traditional datasets.

### 4.2. Target Object Detection

**Figure 11:**
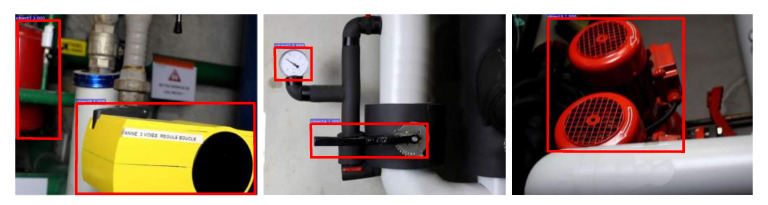
Object detection results (shown in red rectangles) using the fine-tuned Faster R-CNN [46].

Built on the target object detector, the pre-trained pose regression models can be easily switched based on the detected object. For this, we train a Faster R-CNN [46] using the additional collected and annotated images. For each object, there are 1000 and 300 images used for training and verification, respectively. For target object annotation, the popular annotation tool LabelMe [47] is used. We did not train the detector from scratch since the industrial objects are mostly rigid and textureless, and they resemble some objects from ImageNet. For this, the pretrained ImageNet model with the lightweight ResNet18 [48] backbone network was selected, while the other parameter settings introduced in [46] were kept. After 20 epochs, some detection results using the trained model and testing data are presented in Figure 11. We evaluated the detector’s Average Precision (AP) on the testing images of each object and the results are presented in Table 1. We also calculated the mean Average Precision (mAP) by averaging the detection APs across the 10 objects. The mAP was around 99.98%, which is comparatively higher than the reported results in [46]. This is because most objects’ surroundings in industrial environments are much simpler than images taken from natural environments such as in PASCAL VOC [49] and MS COCO [50]. Moreover, objects are more distinguishable from their backgrounds in industrial environments since they normally have different paint surfaces and specular effects. Considering the number of training images and the performance we achieved, the training and testing images for pose regression could also be annotated for training the object detector in practice.

### 4.3. Ablation Studies of WatchPose

**Figure 12:**
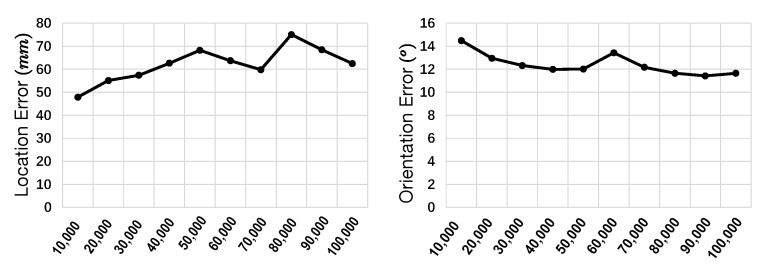
Estimated camera location (mm) and orientation (°) errors using different β values (x axis). β= 40,000 was finally selected for our experiments.

To fully assess the proposed Watchpose scheme and its properties, we performed a set of experiments by fixing the pose regressor to PoseNet [5]. Specifically, we first evaluate and compare the trained PoseNet using original images from both Industrial10-Traditional and Industrial10-WatchPose. The main purpose is to show the performance improvement using our proposed method against the traditional method of collection. We also evaluated the usability of our proposed data augmentation strategy on both datasets. It should be noted that the β value in PoseNet’s loss function should be calibrated based on the scenario. As reported in [6], different β brings considerable performance gaps. Based on the preliminary experiments and discussion in [5], β is mainly influenced by the camera location unit and the scene type (i.e., indoor and outdoor). Since our locations are labeled in millimeter (mm) scale, as compared to the original PoseNet experiments in [5], the computed Euclidean loss values are normally much bigger than the one from orientation. Thus, we systematically searched for appropriate β values by ascending order. Figure 12 illustrates some pose estimation results with different β values using a subset of Industrial10-WatchPose training and testing data (for ease of experimentation). As mentioned in [6], the balanced choice of β must be struck between the orientation and translational errors, both of which are highly coupled as they are regressed from the same model weights. Therefore, we considered both location and orientation estimations and finally selected β = 40,000 for the experiments below.

#### 4.3.1. Original Images

Here, we compare the pose regression performance using the original training data from Industrial10-WatchPose and Industrial10-Traditional. In other words, the images were not augmented. The mean results of each object are detailed in Table 2. We can clearly observe that our proposed WatchPose scheme significantly improved the pose regression performance in this challenging dataset by around 4.7 times for location error and 3.9 times for orientation error, compared to the traditional data collection approaches [5,7].

**Figure 13:**
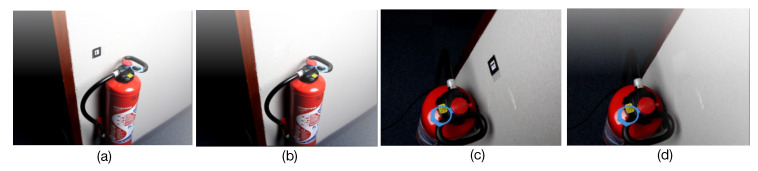
Sample images before (**a**,**c**) and after inpainting (**b**,**d**).

Based on Industrial10-WatchPose, we also experimentally verified the necessity and usability of image inpainting for pose regression in our scenario. Sample images before and after inpainting are shown in Figure 13a,c and b,d, respectively. Particularly, we applied experiments with cross combinations of with-inpainting and without-inpainting for training and testing. Table 3 presents the mean pose estimation results among 10 objects. We can clearly find that with-inpainting training and testing as well as without-inpainting training and testing were similar to each other (around 60∼70 mm location error and 20° orientation error), but both apparently outperformed the other combinations (more than 150 mm location error and 5° orientation error). As in practice the markers were removed after pose generation as it is necessary to apply the inpainting process on training images to meet a promising performance. The followed experiments were all performed based on marker inpainted training and testing images.

#### 4.3.2. Augmentation

In this section, we experimentally explore the efficacy of the data augmentation strategy introduced in Section 3.3 on the Industrial10-WatchPose and Industrial10-Traditional datasets. Following the rotation augmentation introduced in existing reports [6,39], all images were first rotated 4 times. After that, we empirically enriched each training data 4 times within each camera distance interval. For instance, augmenting 4 times within the 0.5 m∼1.0 m camera distance interval meant that the camera distances 0.6, 0.7, 0.8, and 0.9 m were imitated by performing zooming-in and center cropping from the 1.0 m image. Evaluations were done on the same testing data as in Section 4.1 and the result are detailed in Table 4.

We can observe that the improvement of pose regression accuracy using augmented Industrial10-Traditional was limited, around 4%. In contrast, the pose regression accuracy improved around 5.3 times in location and 4.2 times in orientation using the augmented Industrial10-WatchPose. The main reason was that the uncovered regions by the traditional approach were still not properly covered after augmentation. As a result, the performance gap between the traditional and proposed WatchPose approaches was enlarged after data augmentation. Specifically, we achieved errors of around 13.3 mm for location and 4.7° for orientation using Industrial10-WatchPose after augmentation, which is around 19 times better than Industrial10-Traditional.

**Figure 14:**
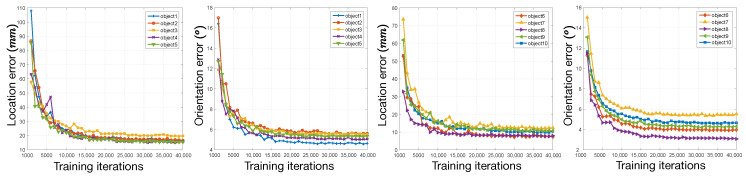
Estimated camera location and orientation errors of 10 objects in each training iteration.

In Figure 14, we plot the pose estimation results in each iteration during the training process using augmented Industrial10-WatchPose. Horizontal axis represents the iteration numbers and vertical axis represents the estimated median performance with respect to camera location and orientation. To optimise the visualisation effect, results from 5 objects are plotted together in each sub-figure. We can clearly observe that both location and orientation errors dramatically dropped after 10,000 training iterations and then stabilized after around 15,000 training iterations.

**Figure 15:**
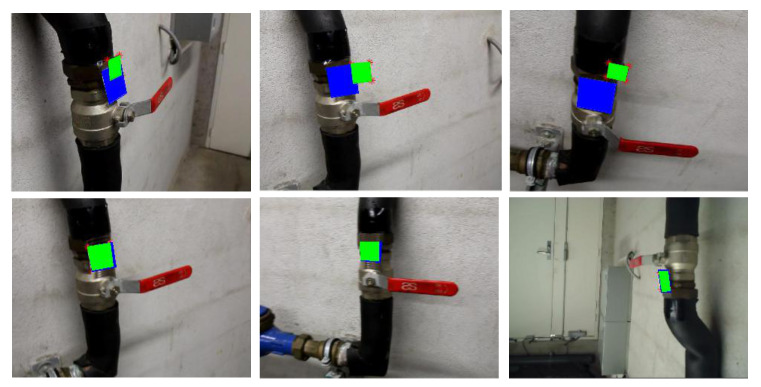
Comparison of marker reprojections (green) between the Industrial10-Traditional (upper) and Industrial10-WatchPose (lower) trained PoseNet. The ground truth is marked in blue.

In addition to the quantitative comparison, we also compare the pose estimation results based on the marker-reprojection approach (some samples shown in Figure 15) so that we can visually observe the differences in performance. For better visualization, we employed the original images before marker inpaining and transferred the relative camera pose of the object back to that of the Nestmarker. If an estimated pose is closer to the ground truth (blue box), the reprojected marker (green box) covers the physical location more accurately. Promising reprojections shown in the lower row of images in Figure 15 demonstrate the robustness of WatchPose. As shown in the upper row, we find that the estimated poses trained with Industrial10-Traditional performed less satisfactorily. The main reason is that the Industrial10-Traditional did not cover enough regions around the target objects thereby the trained PoseNet model could not generalize well on the testing data from uncovered regions.

#### 4.3.3. Dense Control

In this section, we quantitatively evaluate the influence of viewpoint dense control parameter λ in Section 3.3 to the final pose regression performance. To this end, we employ Object1 (λ=0.2) in the Industrial10-WatchPose dataset for the experiment. We also fix other factors such as β in PoseNet, the augmentation strategy, and the testing data. With the increase of λ value (from 0 to 1), original viewpoints of Object1 were proportionally and randomly selected from the virtual ball to imitate the data collection from dense to sparse. For λ=0, 20% of the original viewpoints were randomly selected and duplicated to imitate the overlapped views. The corresponding viewpoints of original images were selected (or duplicated) to generate different training sets. Table 5 presents the pose estimation results with different λ values (the original number of training images before augmentation is also provided for reference). We observed that the trained model performed less and less satisfactorily when λ increased. This is expected since more and more regions were not covered by the training data. We also found that both orientation and location errors were surprisingly slightly higher at λ=0, compared to the performance at λ=0.2. The main reason is that in some regions the viewpoints were densely distributed and redundant with λ=0. As a result, the trained model was slightly overfitted to these regions.

Based on the evaluation in Table 5, we can observe that λ is important to balance the coverage and redundancy of viewpoints. In practice, λ is configured empirically depending on the target object size and the collection conditions. If none of them can be determined in advance, λ=0 is a reasonably acceptable choice of value to ensure the sufficient coverage of different viewpoints, though redundant training images could be generated with this value.

### 4.4. Deep Absolute Pose Estimators

Using the augmented Industrial10-Traditional and Industrial10-WatchPose datasets, we compare the pose regression performance of five existing approaches. Specifically, Bayesian-PoseNet [39] was released by the same authors of the PoseNet approach. Bayesian-PoseNet first generates some samples by dropping out the activation units of convolutional layers of PoseNet based on a probability value. The final pose is then computed by averaging over the individual samples’ predictions. Meanwhile, PoseNet+ [6] introduced a loss with learned uncertainty parameters (learnable weights pose loss) for optimizing PoseNet. Hourglass-Pose [8] focused on optimizing the architecture of PoseNet by suggesting an encoder-decoder architecture implemented with a ResNet34 [48] encoder (removing the average pooling and softmax layers). In contrast to the aforementioned approaches, a more recent method called MapNet [7] suggested to include additional data sources in order to constrain the loss. It is trained with both absolute and relative ground truth data.

On the Industrial10-Traditional dataset, we analyzed the estimation performance of each object category so that deeper observations could be made. The final results are detailed in Table 6. We found that the performances varied among different objects. We also observed that the improved optimization of loss in PoseNet+ and the architecture design in Hourglass-Pose both improved PoseNet’s accuracy by around 2 times, particularly in the location errors which were dramatically reduced. Overall, MapNet achieved the best performances, around 3 times better in location and 2 times better in orientation, compared to the poses estimated by the original PoseNet.

In contrast to Table 6, the estimation errors in Table 7 are smaller among 5 approaches using the Industrial10-WatchPose dataset. This is because the WatchPose data covers more camera poses thereby leading to better generalization in pose regression models. In particular, while the Bayesian-PoseNet only marginally improved the pose regression accuracy (over PoseNet), the Hourglass-Pose and PoseNet+ approaches achieved around 14% improvement in location and 4% in orientation estimation. Once again, MapNet achieved the best performance, with around 226.187 mm in location error and 57.551° in orientation error. It also shows that the proposed WatchPose method of collecting data enabled much higher in both location and orientation performance improvements on MapNet compared to the traditional data collection method. This shows that the proposed protocol is particularly suitable for complex industrial applications as the efficacy of the pose estimation methods are wholly improved.

### 4.5. Discussion on Restrictions

**Figure 16:**
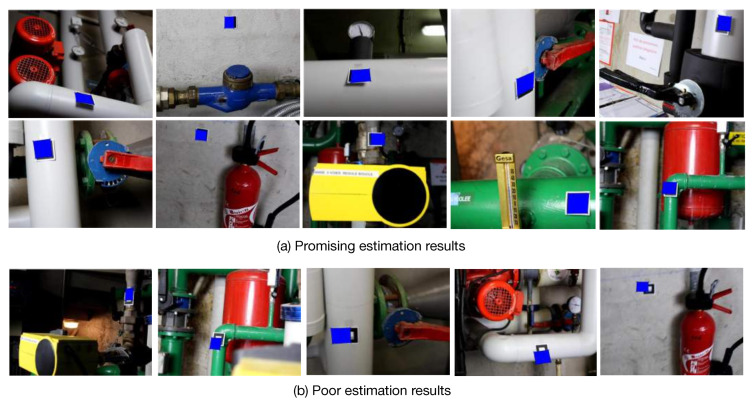
Marker reprojection results (blue) using the estimated camera poses from MapNet [7]. For better visualization, the original marker is attached in each image.

Theoretically, WatchPose is applicable to a scenario when it fulfills the following conditions: (1) Inspections are carried out from within 0.2 to 2 m of the target object. Otherwise, the Nestmarker cannot be properly tracked. (2) There should be enough space around the target object for data acquisition via a handheld camera. (3) There should be a homogeneous place close to the target object for pasting Nestmarkers. However, there could be more factors that influence the efficiency of WatchPose and the performance of pose regression in the application phase. To explore these factors, we employed the marker reprojection approach (similar to Figure 15) so that the pose estimation performance of MapNet could be visually observed, as shown in Figure 16. We can see that most reprojected markers (in blue) on most objects could cover the ground truth (the original marker) quite accurately. However, there were still some testing images with poor reprojection results, as shown in Figure 16b. These images were mainly affected by specific types of challenging conditions: Under- and over-exposed images, partially blurred regions, incorrectly detected objects, and also lesser training data. This is partly attributed to the lighting conditions of the industrial environment. Other factors such as camera configurations and moving speed, shaking, and camera resolution could also impact the image quality and the regression performance. In practice, a camera with high resolution and fixed exposure time is recommended. During the capturing process, the camera should also be moved as slow as possible. Moreover, some tools such as handheld gimbals [51] could be used to stabilize the acquisition. In Section 5, we also introduce two future works to deal with these challenging problems.

Our experiments were performed on two platforms: A laptop and a desktop machine. Training images and camera pose labels were processed on a laptop with Intel Core i7 2.9 GHz CPU, 16 GB installed memory and 64-bit Windows 7 OS. An ELP 1080P HD Webcam was connected to the laptop via a 2 m USB-cable for data collection. Model training, object detection, and pose estimation experiments were accomplished on the desktop machine with 6 Intel Xeon Core 3.5 GHz CPUs, 64 GB installed memory, a Quadro M4000 GPU (8 GB global memory and 1664 CUDA Cores), and Ubuntu 14.04 LTS. The pose generation was implemented with C++. The object detection, pose training, and estimation tasks were implemented with Python. For object detection, the entire training process took around 26 h and the detection (at inference time) took about 0.28 seconds per testing image. For camera pose estimation, each camera pose could be regressed in about 5 to 100 ms depending on the model, which puts the system at real-time speeds.

## 5. Conclusions

In this paper, we introduced a simple but efficient data collection method for complex industrial environments named WatchPose so as to learn effective absolute camera pose regression models. The proposed method integrated the advantageous properties of marker tracking and viewpoint visualization approaches. The features of WatchPose could properly handle the challenges of industrial-type objects: Textureless and specular surfaces under strong artificial lights, and highly variable distances and views angles. We also proposed two post-processing steps (inpainting and augmentation) to improve the robustness and stability of the trained models. To advance pose estimation research in industrial environments, we introduced a new challenging dataset, Industrial10, to represent the aforementioned challenges of industrial-type objects and environments. Experiments showed that the proposed WatchPose method could effectively improve the pose regression performance of five widely-used approaches.

In the future, we propose two further directions. Firstly, we will collect more data to cover other kinds of challenges in industrial environments. For instance, we can enrich the training data by varying an image brightness [52] to imitate different lighting conditions. Secondly, we will compare the motion tracking performance between ARToolKit and ARCore [53], which can be applied with and without markers, respectively. Finally, we will release further baselines for the Industrial10 dataset using a few other camera pose estimation methods such as SfM [32], 3D Scene [30], etc. 

## Figures and Tables

**Table 1:** Average precision of detecting object-of-interest on the testing images.

Object	Average Precision	Object	Average Precision
Object1	95.0%	Object6	97%
Object2	99.2%	Object7	99.6%
Object3	100%	Object8	100%
Object4	99.5%	Object9	100%
Object5	100%	Object10	99.5%

**Table 2:** Estimated camera location (mm) and orientation (°) errors of objects using traditional and WatchPose data collection methods.

Objects	Traditional [5,7]		WatchPose		Objects	Traditional [5,7]		WatchPose	
Object1	368.2857 mm	77.0220°	79.1773 mm	19.7309°	Object6	391.6417 mm	79.5715°	82.9850 mm	17.1254°
Object2	350.5098 mm	63.8454°	81.3936 mm	20.2622°	Object7	291.6453 mm	81.2755°	61.2231 mm	23.2668°
Object3	381.9421 mm	80.2673°	84.8384 mm	21.4031°	Object8	237.5793 mm	59.0616°	50.4712 mm	16.5201°
Object4	341.1610 mm	83.6164°	69.9175 mm	20.8298°	Object9	280.1659 mm	81.4738°	56.7338 mm	18.3799°
Object5	355.6238 mm	86.3258°	73.7141 mm	22.0913°	Object10	278.5611 mm	77.3197°	58.4822 mm	19.9535°

**Table 3:** Pose estimation using with-marker/no-marker data for training/testing.

Train Types	Testing Set: With-Inpainting	Testing Set: Without-Inpainting
Training set: with-inpainting	69.8936 mm 19.9563°	210.5763 mm 25.1174°
Training set: without-inpainting	360.1666 mm 31.7290°	60.6378 mm 20.5549°

**Table 4:** Estimated camera location (mm) and orientation (°) errors of each object using models trained on the augmented Industrial10-WatchPose and Industrial10-Traditional.

Objects	Traditional		WatchPose		Objects	Traditional		WatchPose	
Object1	321.4866 mm	69.1536°	16.6907 mm	4.6411°	Object6	389.0706 mm	76.0915°	7.3524 mm	3.9631°
Object2	311.8624 mm	60.1966°	16.5327 mm	5.6037°	Object7	280.2132 mm	79.5342°	12.4772 mm	5.5193°
Object3	360.3298 mm	76.3290°	19.7768 mm	5.5316°	Object8	229.0576 mm	51.5531°	7.9837 mm	3.0772°
Object4	339.3160 mm	80.3916°	15.6313 mm	5.0524°	Object9	266.6300 mm	75.0026°	10.7575 mm	4.3059°
Object5	354.3400 mm	81.3303°	15.3658 mm	5.3537°	Object10	271.8297 mm	72.1473°	10.4555 mm	4.6450°

**Table 5:** Estimated camera location (mm) and orientation (°) errors of Object1 in using training data with different viewpoint density controlled by λ.

λ	Original Images	Orientation Error (mm)	Location Error (°)
0	2367	16.7402	4.6613
0.2	1793	16.6907	4.6411
0.4	1434	29.989	7.2499
0.6	1075	103.663	26.8137
0.8	717	212.5046	47.2115
1	358	325.8071	72.7677

**Table 6:** Estimated camera location (mm) and orientation (°) errors of different pose regression approaches using the post-processed Industrial10-WatchPose dataset.

Traditional	PoseNet [5]		Bayesian-PoseNet [39]		Hourglass-Pose [8]		PoseNet+ [6]		MapNet [7]	
Object1	16.691 mm	4.641°	17.717 mm	3.223°	7.132 mm	2.952°	6.551 mm	2.338°	5.259 mm	2.100°
Object2	16.533 mm	5.604°	15.505 mm	4.213°	11.040 mm	4.041°	11.608 mm	4.084°	9.646 mm	3.568°
Object3	19.777 mm	5.532°	18.740 mm	4.102°	12.183 mm	4.095°	11.953 mm	3.843°	10.676 mm	3.288°
Object4	15.631 mm	5.052°	17.946 mm	3.986°	9.635 mm	3.541°	8.325 mm	3.476°	9.466 mm	3.096°
Object5	15.366 mm	5.354°	14.364 mm	3.585°	10.865 mm	3.193°	12.225 mm	2.778°	9.105 mm	2.405°
Object6	7.352 mm	3.963°	7.311 mm	2.475°	5.803 mm	2.087°	4.285 mm	2.058°	4.882 mm	1.988°
Object7	12.477 mm	5.519°	13.307 mm	3.985°	7.523 mm	3.371°	6.950 mm	3.112°	5.973 mm	3.020°
Object8	7.984 mm	3.077°	8.154 mm	2.603°	5.419 mm	2.677°	5.727 mm	2.539°	3.785 mm	2.395°
Object9	10.758 mm	4.306°	10.587 mm	3.369°	6.276 mm	2.923°	5.785 mm	2.716°	4.308 mm	2.467°
Object10	10.456 mm	4.645°	11.988 mm	3.555°	6.405 mm	3.114°	6.620 mm	2.996°	4.685 mm	2.411°
Mean	13.303 mm	4.769°	13.562 mm	3.510°	8.228 mm	3.199°	8.003 mm	2.99°	6.779 mm	2.674°

**Table 7:** Estimated camera location (mm) and orientation (°) errors of different pose regression approaches using the augmented Industrial10-Traditional dataset.

WatchPose	PoseNet [5]		Bayesian-PoseNet [39]		Hourglass-Pose [8]		PoseNet+ [6]		MapNet [7]	
Object1	321.487 mm	69.154°	315.742 mm	66.544°	295.021 mm	62.792°	284.158 mm	57.688°	260.900 mm	55.758°
Object2	311.862 mm	60.197°	312.967 mm	55.300°	253.023 mm	56.655°	241.538 mm	51.312°	230.533 mm	48.319°
Object3	360.330 mm	76.329°	355.475 mm	71.164°	290.545 mm	70.936°	279.047 mm	72.325°	233.519 mm	64.396°
Object4	339.316 mm	80.392°	341.601 mm	76.356°	295.942 mm	74.763°	280.991 mm	71.527°	239.109 mm	63.830°
Object5	354.340 mm	81.330°	352.981 mm	74.372°	299.807 mm	79.449°	286.456 mm	72.869°	256.954 mm	57.061°
Object6	389.071 mm	76.092°	265.340 mm	72.558°	322.610 mm	73.795°	310.644 mm	69.460°	269.646 mm	56.306°
Object7	280.213 mm	79.534°	282.665 mm	77.425°	261.754 mm	79.773°	250.111 mm	77.497°	233.322 mm	67.701°
Object8	229.058 mm	51.553°	221.250 mm	56.541°	210.119 mm	49.858°	190.307 mm	47.544°	155.271 mm	40.131°
Object9	266.630 mm	75.003°	275.934 mm	75.422°	215.861 mm	71.598°	203.967 mm	69.300°	181.857 mm	57.695°
Object10	271.830 mm	72.147°	268.961 mm	71.184°	234.627 mm	72.644°	222.443 mm	70.313°	200.762 mm	64.317°
Mean	312.414 mm	72.173°	299.292 mm	69.687°	267.931 mm	69.226°	254.966 mm	65.984°	226.187 mm	57.551°
